# Theogallin-to-Gallic-Acid Ratio as a Potential Biomarker of Pu-Erh Teas

**DOI:** 10.3390/foods12132453

**Published:** 2023-06-22

**Authors:** Kaja Karwowska, Katarzyna Kozłowska-Tylingo, Magdalena Skotnicka, Maria Śmiechowska

**Affiliations:** 1 Division of Food Commodity Science, Faculty of Health Sciences with the Institute of Maritime and Tropical Medicine, Medical University of Gdańsk, ul. M. Skłodowskiej-Curie 3A, 80-210 Gdańsk, Poland; 2 Department of Pharmaceutical Technology and Biochemistry, Faculty of Chemistry, Gdańsk University of Technology, 80-233 Gdańsk, Poland; 3 Department of Quality Management, Faculty of Management and Quality Science, Gdynia Maritime University, 81-225 Gdynia, Poland

**Keywords:** authenticity, theophylline, GS-mass identification, cluster analysis

## Abstract

There are two types of Pu-erh tea available on the world market: Raw and Ripe. It is not difficult to tell them apart if the Raw version is relatively young. Researchers have already developed various tools to identify Pu-erh teas. However, they are quite complicated and require advanced statistical analyses. In addition, they are characterized by different levels of accuracy. The aim of the work was to identify relationships or differences that would easily give specific results for identifying types of Pu-erh tea. The content of selected methylxanthines was determined by high-performance liquid chromatography (HPLC) on an Agilent 1200 chromatograph with a UV-VIS diode array detector. The total analysis time was 28 min. A combination of liquid chromatography and a triple quadrupole mass spectrophotometer was used to identify gallic acid and theogallin in the analyzed samples. A multivariate cluster analysis was used to compare the results for single samples, and its results were presented in horizontal hierarchical tree plots. The quantitative determination showed that theophylline is present only in Ripe Pu-erh teas. In addition, it was shown that the ratio of theogallin to gallic acid can be an effective tool to verify the authenticity of Pu-erh varieties.

## 1. Introduction

**Figure 1:**
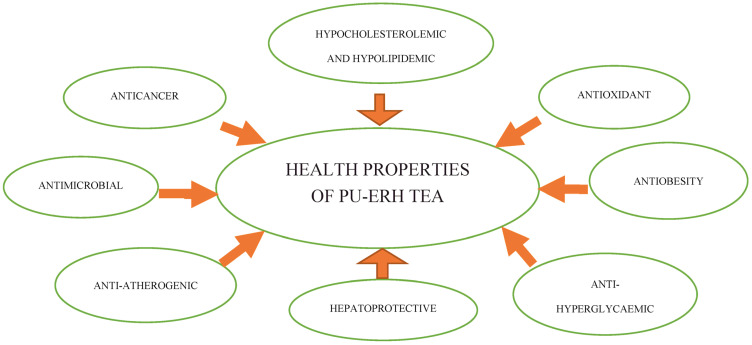
Health properties of Pu-erh tea. Source: own elaboration based on [1,2,3].

Originating from the Yunnan Province of China, Pu-erh tea is gaining more and more importance among other types of Chinese tea due to its unique health properties. In traditional Chinese medicine, Pu-erh tea has been used for centuries to treat metabolic diseases [1]. The results of scientific research confirm its exceptional health properties (Figure 1).

There are two types of Pu-erh: Raw (*Sheng*) and Ripe (*Shu*). Raw Pu-erh is processed like typical green tea and is subject to natural ageing during storage. Ripe Pu-erh differs from Raw Pu-erh in an additional stage—artificial ageing, known as “Wo Dui”, which is introduced to shorten the production process. These differences in the technological processing of raw materials give rise to products with different chemical and sensory characteristics [4]. Traditional Pu-erh is Raw tea subject to long-term ripening—after which it is called aged Raw Pu-erh. The best tea products of this type are not available for retail sale, but they are sold during auctions, at enormous prices (for thousands of dollars). Therefore, a great opportunity emerges to leverage earnings unlawfully by selling counterfeit products [5]. 

The differentiation between Raw and Ripe Pu-erh is not difficult if the Raw version is relatively young. However, the difference between aged Raw and high-quality Ripe Pu-erh is not that obvious. The confirmation of the specific tea’s origin, year, and type is the responsibility of tea masters (tea sommeliers). It is possible that when a unique Pu-erh product is launched on the market, the knowledge and, above all, the experience of new experts turn out to be insufficient to confirm that the presented product is genuine. Although those involved in tea auctions do not deem this necessary, scientists have been pursuing an unfailing method for identifying various types of tea, determining the origin of the raw material, and estimating the age of tea.

Like other products, tea’s origin, variety, and even harvest time can be identified. In addition, multiple attempts have been made to design tools for efficiently identifying various types of tea and their origin [6,7,8,9,10]. Although Raw and Ripe Pu-ehr are completely different and easy to discriminate immediately after the production process, estimating the age of aged Raw and discriminating between aged Raw and good-quality Ripe Pu-erh seems a more difficult task. In addition, for regional products, the origin of raw materials is less varied. 

Therefore, scientists use different research methods and look for new tools to distinguish between these two main types of Pu-erh tea. Ku et al., (2010) [11] used metabolomics to analyze the types of Pu-erh tea and changes in the composition of the product depending on its maturation time. In addition, they showed that Raw Pu-eh contained more antioxidants, thanks to which its antioxidant activity (as measured by the TEAC, DPPH, and FRAP tests) was higher than that of the Ripe version. Lv et al., (2014) [12] studied the methoxyphenol composition of “Dayi” Pu-erh and designed a chromatographic “fingerprint” (characterization of aromatic compounds), which, in combination with chemometric analysis, can be used in the identification and quality control of this tea. The research of Cao et al., (2018) [13] shows that volatile organic compounds (VOCs), such as methoxyphenols, can be markers of this type of tea. One of the directions of research is also to determine the origin of Pu-erh tea. Such studies were conducted, among others, by Wu et al., (2016) [14], who found that the content of some terpene alcohols and ketones varied significantly depending on the origin of the product.

Despite scientists having developed several effective tools for assessing the quality of Pu-erh tea, none of the proposed solutions has been approved on the auction market due to complex procedures and high cost. Thus, new reliable research methods are being sought for simpler and less expensive Pu-erh tea quality assessment and authenticity testing (identity check). Therefore, an attempt was made to identify the relationships or differences that would easily provide certain outcomes for the identification of Pu-erh tea types. In the first part of this research, the content of selected methylxanthines in Raw and Ripe Pu-erh teas was determined. Methylxanthines such as caffeine, theobromine, and theophylline are common compounds in tea, but there is no information about their use as biomarkers in identifying types of Raw and Ripe Pu-erh tea. The second part concerned the determination of the content of gallic acid and theogallin in Raw and Ripe Pu-erh teas. In this case, we also wanted to test the use of these compounds as biomarkers to distinguish types of Raw and Ripe Pu-erh tea.

## 2. Materials and Methods

### 2.1. Plant Materials

Tea samples were purchased from Chawang Shop in Yunnan province, Kunming City, China, and delivered to Poland by air. The study involved five samples of Raw and five samples of Ripe Pu-erh tea of different ages and origins.

### 2.2. Chemicals and Reagents

Gallic acid (3,4,5-Trihydroxybenzoic acid, CAS Number 149-91-7) and selected methylxanthines were purchased from Sigma-Aldricht (Schnelldorf, Germany): theobromine (3,7-Dimethylxanthine, CAS Number 83-67-0), theophylline (1,3-Dimethylxanthine_,_ CAS Number 58-55-9), and caffeine (1,3,7-Trimethylxanhine, CAS Number 58-08-2). In turn, theogallin (3-Galloylquinic acid, CAS Number 17365-11-6) was supplied by MolPort (Riga, Latvia).

### 2.3. HPLC-DAD-MS Analysis

The content of selected methylxanthines was assayed through high-performance liquid chromatography (HPLC) in an Agilent 1200 chromatograph with a UV-VIS diode array detector (Agilent Technologies, Waldbronn, Germany). A satisfactory chromatographic separation was obtained for a mobile phase composed of methanol/water/formic acid (Sigma-Aldricht, St. Louis, MO, USA) in the proportion 19.9%/79.8%/0.3% *v*/*v* in an isocratic run at a flow rate of 1 mL/min. The analysis was conducted using the Intersil ODS-3 column (5 μm, 250 mm × 4.6 mm, GL Sciences Inc., Tokyo, Japan), and the sample injection volume amounted to 20 μL. All assays were performed at the temperature of 23 °C, measuring radiation absorbance at wavelength 273 nm. The total analysis time was 28 min.

Using a combination of liquid chromatography (LC set 1260 Infinity II from Agilent Technologies, Waldbronn, Germany) and a triple quadrupole mass spectrophotometer (6470 Triple Quad LC/MS, Agilent Technologies, Santa Clara, CA, USA), the presence of gallic acid and theogallin was identified in the tested samples. The mass spectra of ions (*m*/*z*) were obtained through SCAN.

### 2.4. Sample Preparation

The tea was ground with a grinder (Moulinex 980) for 10 s. Afterwards, 2 g of tea was weighed (with an accuracy of up to 0.0001 g) and poured with 100 mL of demineralized water at a temperature of 98 °C ± 2 °C. All teas were brewed under cover for 15 min. In the end, the infusions were filtered through 0.22 mm drains. 

### 2.5. Data

Three replications of independently prepared samples were run for each tea (standard deviation (SD) range of 0.3–1.0%). The results were presented as mean values. Significance of differences was demonstrated by analysis of variance at a significance level of *p* ≤ 0.05 and Fisher’s post hoc test.

Multivariate cluster analysis was applied to compare the results for single samples, and its results were presented on horizontal hierarchical tree charts. The clusters were determined by applying Euclidean distance to the similarity scale and using Ward’s linkage method. Cluster analysis was used on account of an intelligible grouping of the examined teas into relatively uniform classes based on similarities between the analyzed samples. The results were elaborated on using the PQStat program from PQStat Software (version 1.4).

## 3. Results and Discussion

### 3.1. Assaying Selected Methylxanthines

The results (Table 1) imply that theophylline can constitute a biomarker of Pu-erh tea, allowing for discrimination between Raw and Ripe tea. This compound was absent from all samples of the traditional Raw tea. By contrast, Ripe tea samples contained from 1.2 to 31.2 mg of theophylline per 100 g of the product. Chen et al. [2] identified the highest GA content in a sample stored for 27 years, i.e., 10.12 mg/g, while in a sample stored for 75 years, it was only 0.13 mg/g. The content of gallic acid seems to be very important, as Chen et al. [2] showed that its content was correlated with anticancer properties by influencing the proliferation of human hepatocellular carcinoma.

However, other authors did not corroborate such a relationship. Lin et al., (1998) [15] identified theophylline only in three (0.06–0.10 mg/100 mg of dried leaves) out of seven of the analyzed Ripe Pu-erh samples. Kłódka, Bońkowski, and Telesiński (2008) [16] did not find it in any of the analyzed Pu-erh infusions (refers to Ripe Pu-erh). In turn, Zhao et al., (2011) [17] identified theophylline in green Pu-erh (at amounts smaller than 0.2 mg/g), while Wang et al., (2018) [18] did not find theophylline in two out of three analyzed samples of commercial instant Ripe Pu-erh. Therefore, theophylline cannot be used for identifying the type of Pu-erh, and the observed relationship should be deemed a coincidence stemming from the selection of the analyzed material. Raw material processing does not determine the content of the analyzed compound. 

Primarily, the level of theophylline in the final product is determined by its content in the raw material, which is closely linked with the content of caffeine and the species of microorganisms present during fermentation. Yang et al., (2007) [19] demonstrated that *Camellia* genus plants (*Camellia sinensis*, *Camellia ptilophylla*, and *Camellia assamica* var. *kucha*) contain purine alkaloids (caffeine, theobromine, theophylline, theacrine, adenine, xanthine, hypoxanthine, and paraxanthine) and a small share of pyrimidine alkaloids. The main alkaloid derived from dry leaves of *Camellia sinensis* and *Camellia assamica* is caffeine (ca. 2–5%). It is produced mainly by young leaves, where it is continually accumulated as the leaves ripen, simultaneously being slowly degraded. The main caffeine biosynthesis pathway in a tea plant is xanthine being converted into caffeine by 7-methylxanthosine, 7-methylxanthine, and theobromine. The rate of caffeine biosynthesis depends on the amount and activity of enzymes, available substrates, specific tissue development stages, growth period, and environmental conditions. Caffeine in plant roots is a result of the three-stage methylation of xanthosine. In contrast, during the catabolism of caffeine in a tea plant, theophylline is only a transient metabolite, and its level remains very low since, in this process, caffeine is degraded into xanthine by theophylline and 3-methylxanthine, which are further degraded through the conventional catabolism of purine into CO_2_, NH_3_ and urea by uric acid, allantoin, and allantoic acid [20,21,22]. 

The specific process of fermentation of Pu-erh teas (notably Ripe Pu-erh) is important for determining the level of caffeine and theophylline in the final product. Knowledge of the influence that respective microorganisms may have on the content of various compounds can be used for producing tea with a specific composition. Wang et al., (2008) [23] made use of selected genera of molds and yeasts (*Aspergillus niger*, *Rhizopus arrhizus*, *Mucor circinelloides*, *Candida albicans*, and *Candida famata*) in the fermentation of tea, during which the levels of caffeine, theobromine, and theophylline were measured. They demonstrated that mold fermentation increased caffeine content, while yeasts contributed to its decline. *Aspergillus niger* was the strain with the strongest influence on caffeine content, increasing from initially 3.47% to 9.63%. Changes in caffeine and theophylline showed a similar trend. Scientists found that caffeine in the fermented product derived from methylation of theophylline, which occurred during microbial metabolism. By contrast, Zhou et al., (2018) [24] isolated 11 strains of fungi from Pu-erh and investigated how they affected caffeine levels during fermentation. They demonstrated that mainly *Aspergillus niger* and *Aspergillus sydowii* were capable of degrading caffeine, and its main decomposition products were theophylline and 3-methylxanthine. Their levels significantly increased as the degradation of caffeine was progressing. In their next study, Zhou et al., (2019) [22] found that caffeine is concurrently demethylated and oxidized during secondary microbial metabolism—demethylation being the main pathway. A product of the decomposition of caffeine by fungi is mostly theophylline. *Aspergillus niger*, *Aspergillus pallidofulvus*, *Aspergillus sesamicola*, and *Penicillium mangini* significantly increased caffeine content but did not affect theophylline levels. The use of *Aspergillus sydowii* in the process of fermentation considerably decreased the content of caffeine (from initially 34.39 mg/g to 5.54 mg/g on the 15th day of the process) and concurrently increased the level of theophylline (from initially 0.43 mg/g to 25.03 mg/g on the 15th day of the process). About 93% of the degraded caffeine was converted into theophylline. In other studies, Zhou et al., (2020) [25] isolated seven species of fungi from Pu-erh that are responsible for the decomposition of theophylline. In particular, *Aspergillus ustus* and *Aspergillus tamari* considerably degraded theophylline into 3-methylxanthine and xanthine through N-demethylation and oxidation. 

**Figure 2:**
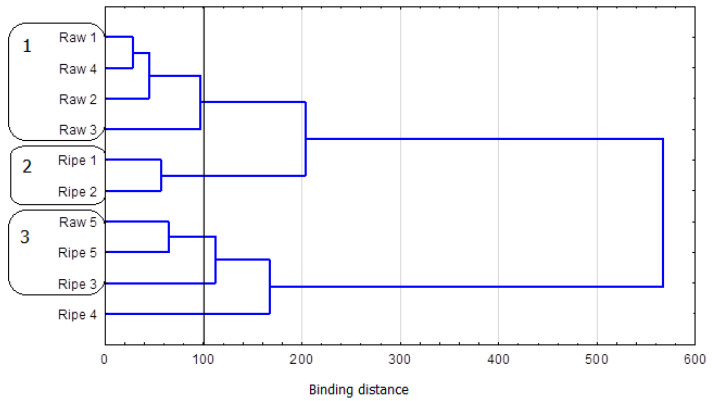
Horizontal hierarchical tree chart of the cluster analysis of selected variables. Grouping of types of tea according to the content of selected methylxanthines.

Other assays we performed do not imply any other relationship which could be used for confirming that Pu-erh teas are genuine. In the Raw Pu-erh group, younger teas contained more theobromine than their older equivalents. Thus, it is possible that when the tea-ripening time is longer, the content of theophylline will decrease, and, in turn, the level of caffeine will increase (the relationship between the content of theophylline and caffeine is determined by the above-described conversions). However, the existence of such a relationship needs to be corroborated by further studies. The results for the total content of theobromine, theophylline, and caffeine also do not reveal the existence of a relationship that could be used as a tool for the identification of Pu-erh tea types. Thus, we decided to verify whether grouping based on three parameters in the multivariate cluster analysis would show other relationships between the evaluated samples. Figure 2 presents three clusters according to the binding distance for respective stages at an agglomeration distance of 100.

The multivariate cluster analysis demonstrated that Pu-erh teas could not be effectively classified using the total content of caffeine, theophylline, and theobromine as the classification criterion. The first cluster is formed by Raw 1 and Raw 4 (Euclidean distance = 28) and by Raw 2 and Raw 3. These teas do not contain theophylline, but they contain similar amounts of caffeine, and theobromine content is among the highest measured levels. Ripe 1 and Ripe 2 form another cluster (Euclidean distance = 56). Out of all Ripe Pu-erh teas, these two samples had the highest content of theobromine and theophylline, and the caffeine level was similar. The third identified cluster comprised the youngest sample of Raw Pu-erh from 2014 and two samples of Ripe Pu-erh (from 2007 and 2015). In that cluster, teas showed varying levels of the assayed methylxanthines: theobromine, from 19.9 to 43.6 mg/100 g product; theophylline, from “not detected” to 13.4 mg/100 g product; and caffeine, from 382.6 to 511.0 mg/100 g product. At the agglomeration distance selected for the analysis, one of the Ripe Pu-erh samples was outside the identified clusters. Ripe 4 from 2010 had one of the lowest contents of theobromine and theophylline and the highest level of caffeine (588.2 mg/100 g product). 

The presented grouping illustrates the high diversity of the analyzed material in terms of the content of selected methylxanthines, thus disqualifying this analysis from successfully using such compounds for the identification of Pu-erh types.

### 3.2. Assaying Gallic Acid and Theogallin

**Figure 3:**
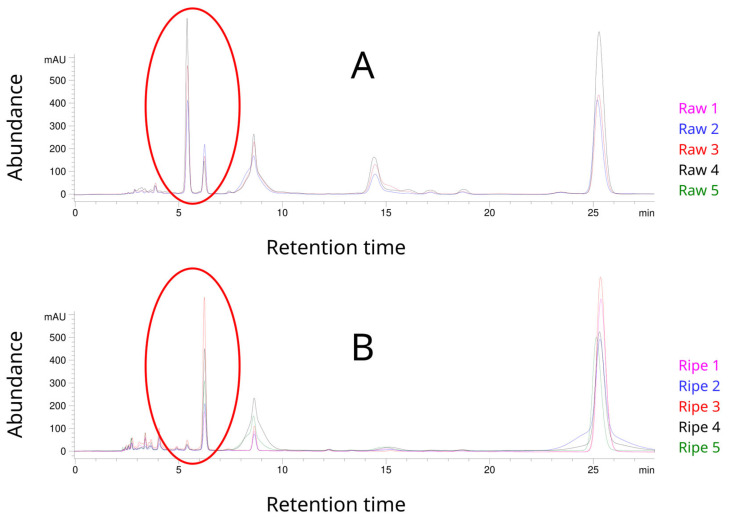
The relationship between peaks at 5.4 and 6.2 min in individual groups of the tested material: (**A**) Raw Pu-erh tea and (**B**) Ripe Pu-erh tea.

During a visual assessment of chromatograms, a potential relationship was observed between two peaks occurring at 5 min 40 s and 6 min 20 s. For traditional Pu-erh, both peaks were clearly marked, and the first was higher than the second one. In contrast, the first peak was definitely lower than the second one for Ripe tea (Figure 3).

The chromatographic analysis, combined with mass spectrometry, showed that the first identified substance was theogallin, and the second was gallic acid. The assays revealed that traditional Pu-erh contained several times more theogallin than gallic acid. In turn, an opposite relationship was found in Ripe Pu-erh—the level of gallic acid was several times higher than that of theogallin (Table 2).

The samples of traditional Pu-erh contained one of the highest assayed levels of theogallin. We found that older samples contained less theogallin in that group than younger samples. In turn, the content of gallic acid increased with the age of tea, but this ended with Raw Pu-erh from 2003 which contained the highest amount of this compound (165.4 mg/100 g product). The oldest sample from 1998 contained 148.3 mg of gallic acid per 100 g of the product. Ripe teas contained several dozen times less theogallin than Raw teas did. In contrast, the gallic acid content in the analyzed group of Ripe Pu-erh teas was, on average, higher than in Raw teas. Younger Ripe Pu-erhs (2015–2017) contained substantially more gallic acid than the oldest sample of Ripe and all Raw Pu-erhs.

Based on these findings, it can be stated that the theogallin-to-gallic-acid ratio can be used as a tool for the identification of Pu-erh tea types. Its efficiency and reliability, as well as subsequent applications, should be corroborated by further research.

The available reference literature does not directly report any relationship between the content of theogallin and gallic acid. However, we decided to review single results found in some scientific publications. Data presented by Hou et al., (2009) [26] and Pedan et al., (2018) [27] corroborate the observed relationship. However, the first team did not identify gallic acid in non-fermented Pu-erh, and the fermented version did not contain theogallin. In contrast, the second team assayed selected active compounds in Raw Pu-erh (five products from 2005 and one from 1992 and 1980 each) and young Ripe Pu-erh (five products from 2016, five products from 2017, and one from 1994 and 1980 each) of various origin. All the Raw Pu-erhs contained from 2.6 to 11.1 more theogallin than gallic acid, and in all the Ripe tea samples, the assayed content of gallic acid was from 1.2 to 16.6 higher than that of theogallin. Moreover, Wang et al., (2018) [28] assayed the two said compounds in Raw and Ripe Pu-erh and found an identical relationship—with one exception. A quantitative analysis was conducted for samples from 2015, 2012, and 2011. Again, the Raw version of the tea contained several times more theogallin (about 4.3 times) than gallic acid. In Ripe Pu-erh from 2015 and 2012, the content of gallic acid measured was from 1.2 to 1.5 higher than that of theogallin. Such a relationship was not present in the sample from 2011. All Ripe Pu-erhs featured a relatively low content of gallic acid, which, after microbial fermentation, should be substantially higher than in Raw Pu-erh [11,26,29,30]. The high variability of results for Ripe Pu-erh can also give rise to concerns. For this reason, we believe that the abovementioned deviation from the proposed rule does not clearly exclude the possibility of a relationship between the content of theogallin and gallic acid that could be used to identify the type of Pu-erh tea. This can also be corroborated by the results reported by Ge et al., (2019) [29], who determined the characteristics and content of phenolic acids in Pu-erh samples by using a newly developed assay. In their work, the authors highlighted that gallic acid was the main phenolic acid of Ripe Pu-erh, while theogallin was the main one of Raw Pu-erh. 

The relationship demonstrated by other authors and us can be explained by conversions occurring during fermentation. In Ripe Pu-erh, the content of theogallin decreases since microorganisms metabolize it into gallic acid. The gallic acid content also increases due to other conversions (e.g., from EGCG) [29]. 

Theogallin (3-galloyloquinic acid), as an ester of gallic acid, is the most common phenolic acid present in all types of tea. It was demonstrated that, in Japanese green tea, it is one of the compounds enhancing the intensity of the umami taste. Its content differs depending on the species of *Camellia sinensis*, the maturity of leaves, and the harvest time. Theogallin has an antioxidant and anti-inflammatory effect. The body metabolizes it into gallic acid and quinic acid [29,31,32,33]. German scientists demonstrated that theogallin and quinic acid could be an effective preventive treatment, or even a cure, for dementia (e.g., Alzheimer’s or Parkinson’s disease), depression, and attention deficit disorders such as attention deficit hypersensitivity disorder (ADHD). In 2007, scientists patented the application of these two compounds in the production of a therapeutic agent [34,35]. Despite the fact that theogallin is not a newly discovered compound and its presence in tea was intensively analyzed as early as the 1960s and 1980s, the reference literature gives it little coverage.

In turn, gallic acid (3,4,5-Trihydroxybenzoic acid) is a phenolic compound that is commonly found throughout the kingdom of plants. It occurs in a free form or as a phenolic secondary metabolite. Gallic acid is mostly used in the pharmaceutical and chemical industry due to having several important properties. A strong antioxidant, it captures free radicals and reduces the effects of oxidative stress. It also has an anticancer effect (inhibiting cell proliferation and inducing apoptosis), as well as antibacterial, antifungal, antiviral, anti-inflammatory, antidiabetic, and neuroprotective effects, and protects the cardiovascular system [36,37,38]. The content of gallic acid in tea is substantially determined by the technological processing of the raw material. It was demonstrated that the more processed the raw material is, the higher the gallic acid content in the final product. Thus, black and dark teas contain more gallic acid than green and white teas do [15,39]. In Pu-erh, catechin gallates and theaflavins are hydrolyzed by tannase (produced by fungi), releasing gallic acid, catechins (EC, C, GC, and EGC), and theaflavin. Next, catechins are further polymerized or isomerized, and gallic acid is esterified into ethyl gallate [15,38]. It was also found that strictinin disintegrates into ellagic and gallic acid during heating processes in tea production [40]. 

**Figure 4:**
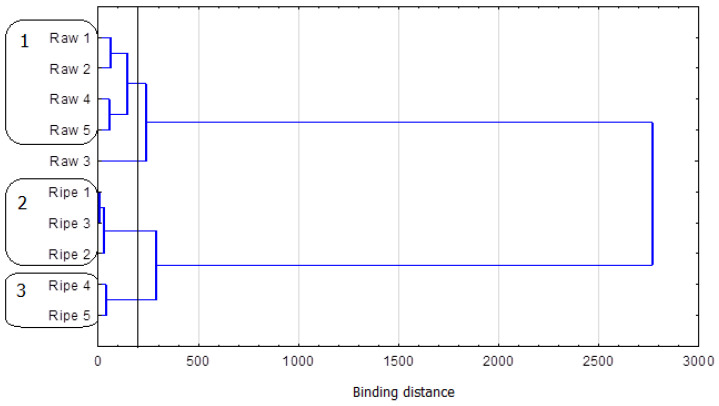
Horizontal hierarchical tree chart of the cluster analysis of selected variables. Grouping of types of tea according to the content of gallic acid and theogallin.

The multivariate cluster analyses produced three clusters (agglomeration distance: 200) (Figure 4).

For the selected agglomeration distance, the first cluster is formed by Raw 1 and Raw 2 (Euclidean distance = 62) and by Raw 4 and Raw 5 (Euclidean distance = 56). Raw 3 from 2007 stood out from other Raw Pu-erhs since it contained the highest assayed level of theogallin in the whole group. Ripe 1 and Ripe 3 (Euclidean distance = 7), together with Ripe 2, formed another cluster. The samples contained more theogallin than two other Ripe Pu-erhs and the highest assayed levels of gallic acid (from 221.0 to 247.2 mg/100 g product). The last cluster was Ripe Pu-erh from 2005 and 2003 (Euclidean distance = 39). These two samples featured the lowest content of theogallin (18.1 and 19.2 mg/100 g product) and the lowest content of gallic acid among Ripe teas (75.1 and 114.5 mg/100 g product). 

The presented grouping for the selected agglomeration distance includes more than two groups of teas. In addition, one of the analyzed samples was not included in any cluster. This could suggest that the analysis of the content of theogallin and gallic acid may not be an efficient tool for the identification of Raw and Ripe Pu-erh tea. Nevertheless, the presented method is 100% efficient for larger agglomeration distances (>300). 

For the analyzed group of teas, it was demonstrated that, in assaying the content of gallic acid and theogallin, no detailed statistical analysis is required, as the ratio between these two compounds is an efficient tool for identifying Pu-erh tea. This is undoubtedly the greatest advantage of the proposed method since it provides clear deliverables that do not require additional elaboration and interpretation.

## 4. Conclusions

The analysis of the content of selected methylxanthines indicated that the presence of theophylline can be treated as a marker of Pu-erh teas. This compound was detected in all Ripe Pu-erhs. It was not detected in the Raw Pu-erh version. However, this relationship has not been observed by other researchers, thus casting doubt on the effectiveness of this tool in identifying the type of Pu-erh tea. These studies should certainly be repeated on more extensive research material. In addition, the chromatographic analysis revealed a potential relationship between the two peaks. They were identified as gallic acid and theogallin. Raw Pu-erh was characterized by a much higher theogallin content compared to Ripe Pu-erh. The latter, in turn, was characterized by a higher content of gallic acid each time than the Raw version. It was therefore found that the analysis of gallic acid and theogallin content, expressed as the ratio of the content of these two substances, with high probability can be successfully used to determine the type of Pu-erh tea. The research was conducted on a small number of teas, therefore, to confirm the effectiveness of this method, further research should be carried out using more diverse research material. This will additionally exclude certain exceptions.

## Figures and Tables

**Table 1:** Content of selected methylxanthines per 100 g of the product.

Type of Pu-Erh Tea	Production Date	Crop Type	Quantity per 100 g of the Product (mg)		
			Theobromine	Theophylline	Caffeine
Raw 1	1998	Arbor tea garden	43.6 ^a^	ND	511.0 ^b^
Raw 2	2003	No data	171.7 ^b^	ND	474.5 ^c^
Raw 3	2007	Arbor tea garden	251.1 ^b^	ND	466.7 ^c^
Raw 4	2012	No data	200.7 ^b^	ND	461.6 ^c^
Raw 5	2014	Old tea trees	152.1 ^a^	ND	454.8 ^b^
Ripe 1	2003	No data	21.6 ^a^	13.4 ^a^	451.9 ^b^
Ripe 2	2005	No data	23.6 ^b^	8.0 ^b^	588.2 ^c^
Ripe 3	2007	No data	19.9 ^a^	1.2 ^a^	382.6 ^b^
Ripe 4	2010	Arbor tea trees	250.6 ^b^	24.1 ^a^	553.1 ^c^
Ripe 5	2015	No data	195.6 ^b^	31.2 ^a^	563.9 ^c^

Data are shown as mean values. Values in the same row within a selected group marked with different letters are statistically significantly different at *p* < 0.05. No data—the seller did not provide this information in the product description. ND—not detected.

**Table 2:** Theogallin and gallic acid content per 100 g of the product.

Type of Pu-Erh Tea	Production Date	Crop Type	Quantity per 100 g of the Product (mg)	
			Theogallin	Gallic Acid
Raw 1	1998	Arbor tea garden	576.1 ^b^	148.3 ^a^
Raw 2	2003	No data	629.5 ^b^	165.4 ^a^
Raw 3	2007	Arbor tea garden	795.1 ^b^	118.2 ^a^
Raw 4	2012	No data	687.3 ^b^	75.9 ^a^
Raw 5	2014	Old tea trees	667.6 ^b^	60.9 ^a^
Ripe 1	2003	No data	18.1 ^a^	75.1 ^b^
Ripe 2	2005	No data	19.2 ^a^	114.5 ^b^
Ripe 3	2007	No data	22.7 ^a^	221.0 ^b^
Ripe 4	2010	Arbor tea trees	27.8 ^a^	247.2 ^b^
Ripe 5	2015	No data	29.0 ^a^	224.9 ^b^

Data are shown as mean values. Values in the same row within a selected group marked with different letters are statistically significantly different at *p* < 0.05. No data—the seller did not provide this information in the product description.

## Data Availability

The data used to support the findings of this study can be made available by the corresponding author upon request.
